# Bloch Surface Waves in Open Fabry–Perot Microcavities

**DOI:** 10.3390/mi14030509

**Published:** 2023-02-22

**Authors:** Niccolò Marcucci, Tian-Long Guo, Ségolène Pélisset, Matthieu Roussey, Thierry Grosjean, Emiliano Descrovi

**Affiliations:** 1 Dipartimento di Scienza Applicata e Tecnologia, Politecnico di Torino, 10129 Torino, Italy; 2 Center for Photonics Sciences, Department of Physics and Mathematics, University of Eastern Finland, 80101 Joensuu, Finland; 3 Department of Optics, FEMTO-ST Institute, UMR CNRS 6174, 25030 Besançon, France

**Keywords:** Bloch Surface Waves, strong coupling, TMD, 2D materials

## Abstract

Thanks to the increasing availability of technologies for thin film deposition, all-dielectric structures are becoming more and more attractive for integrated photonics. As light–matter interactions are involved, Bloch Surface Waves (BSWs) may represent a viable alternative to plasmonic platforms, allowing easy wavelength and polarization manipulation and reduced absorption losses. However, plasmon-based devices operating at an optical and near-infrared frequency have been demonstrated to reach extraordinary field confinement capabilities, with localized mode volumes of down to a few nanometers. Although such levels of energy localization are substantially unattainable with dielectrics, it is possible to operate subwavelength field confinement by employing high-refractive index materials with proper patterning such as, e.g., photonic crystals and metasurfaces. Here, we propose a computational study on the transverse localization of BSWs by means of quasi-flat Fabry–Perot microcavities, which have the advantage of being fully exposed toward the outer environment. These structures are constituted by defected periodic corrugations of a dielectric multilayer top surface. The dispersion and spatial distribution of BSWs’ cavity mode are presented. In addition, the hybridization of BSWs with an A exciton in a 2D flake of tungsten disulfide (WS_2_) is also addressed. We show evidence of strong coupling involving not only propagating BSWs but also localized BSWs, namely, band-edge and cavity modes.

## 1. Introduction

The existence of optical modes strongly confined at the truncation interface of planar dielectric multilayers (one-dimensional photonic crystals, -1DPC) has been known for decades as a result of the pioneering studies by P. Yeh and A. Yariv [1,2] and several later works [3,4,5]. With the advent of the Plasmonics era, interest toward optical surface modes on planar dielectric stacks has seen a significant boost, mainly due to the possibility of overcoming some the inherent limitations of surface plasmons, especially in sensing applications [6,7,8,9]. In the last 20 years, optical surface modes (hereafter called Bloch Surface Waves, or BSW) on flat and patterned dielectric multilayers have been investigated in many frameworks, such as strong light–matter coupling [10,11,12,13], integrated [14,15,16,17,18], guided [19,20,21,22] and fiber [23,24] optics, label-free [25,26,27,28] and fluorescence-based [29,30,31] sensing, metrology, [32] photon management [33,34,35,36], light-driven particle manipulation, [37,38] emitting devices [39,40], microscopy imaging, and spectroscopy [41,42,43].

Besides the advantages of tuning the BSWs’ spectral position, polarization [44], as well as propagation and penetration lengths [45], one of the most intriguing features offered by 1DPC-based platforms is the possibility to exploit the 1DPC surface to set up an accessible framework for controlling light–matter interaction. Furthermore, shallow patterning of the 1DPC surface allows the BSW to be manipulated and possibly confined along transverse directions [40,46], thus providing new degrees of freedom for exploring complex phenomena. Having a photonic mode confined both out-of-plane and in-plane on a surface is advantageous when alleviating some difficulties in technological tasks such as the integration of emitters/absorbers, which are typically buried/grown within the photonic structure itself, where the radiation energy of photonic modes is generally localized.

In this article, we present a computational work describing some peculiar features of transverse electric (TE)-polarized BSWs confined within linear resonant structures recalling Fabry–Perot microcavities. In addition, the interaction of cavity BSWs with the A exciton in a WS_2_ monolayer laying on top of the multilayered structure is investigated and discussed. WS_2_ monolayers display a narrow and intense excitonic resonance at 2.03 eV that is well suited to promote mode hybridization with BSWs. Extending previous results reporting on strong coupling between the WS_2_ exciton and BSWs on flat dielectric multilayers [47], we anticipate here that strong coupling can be also observed with BSWs confined within planar cavities covered by WS_2_ flakes of down to about 1 μm in transverse size.

## 2. Computational Model

**Figure 1:**
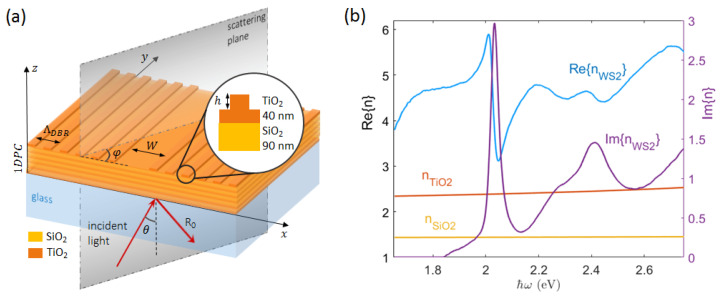
(**a**) Sketch of the patterned 1DPC in the conical diffraction mounting. The topographic modulation is oriented along the x-direction. Light is incident from the glass substrate at an angle of incidence θ; (**b**) refractive index dispersion of the materials considered in this work.

The exemplary model for the 1D photonic crystal considered in this work is constituted by a stack of five alternating pairs of TiO_2_ and SiO_2_ layers on a semi-infinite glass half-space (refractive index $n_{glass}=1.5$). The TiO_2_ and SiO_2_ layers have thickness 85 nm and 110 nm, respectively, so that a main forbidden band is crossing the light line at energies of about 2 eV. On top of the stack, an additional pair of SiO_2_ (90 nm) and TiO_2_ (40 nm) layers is introduced, leading to a total of N_L_ = 12 layers. These two layers are aimed at tailoring the position of the BSW across the forbidden band, in the energy–momentum space. In addition, the upmost TiO_2_ layer is intended as a functional medium carrying a topographic modulation. In fact, a linear grating acting as a Distributed Bragg Reflector (DBR) is engraved therein, with a modulation height h=25 nm, period $\Lambda_{\mathrm{DBR}}=275$ nm, and fill factor 0.5. A linear defect (hereafter called a “spacer”) with a width W is eventually introduced within the DBR, thus resulting in a linear Fabry–Perot cavity. A schematic of the structure is presented in Figure 1a.

As will be apparent from the following, the DBR is aimed at perturbing the BSW dispersion by opening a bandgap at the crossing point of the BSW dispersion with the boundary of the first Brillouin zone defined by the DBR. The bandgap width is determined by the BSW (effective) refractive index contrast associated to the corrugation. Generally speaking, the larger the topographic height modulation, the wider the BSW bandgap. However, practical limitations to the maximum attainable width of the BSW bandgap exist. For example, if the modulation height or the top layer thickness are too large, the dielectric loading/unloading effect may cause BSWs to disappear in either the trenches (low effective refractive index) or the ridges (high effective refractive index), thus preventing a BSW bandgap to open. The geometry proposed here represents a (not unique) solution that avoids the occurrence of such an issue, while allowing the formation of a reasonably wide BSW bandgap for observing cavity BSWs and related hybridization with the WS_2_ exciton.

This kind of dielectric multilayer can be fabricated by means of standard techniques, including RF sputtering [16], chemical vapor deposition (CVD) and plasma-enhanced CVD [48] plasma ion-assisted vacuum evaporation (PIAD) [49], and atomic layer deposition (ALD) [50]. In particular, the refractive indexes used to design the present stack are taken from ellipsometric data collected from ALD-deposited materials and plotted in Figure 1b. In addition, the refractive index of a WS_2_ monolayer is plotted as well [51].

**Figure 2:**
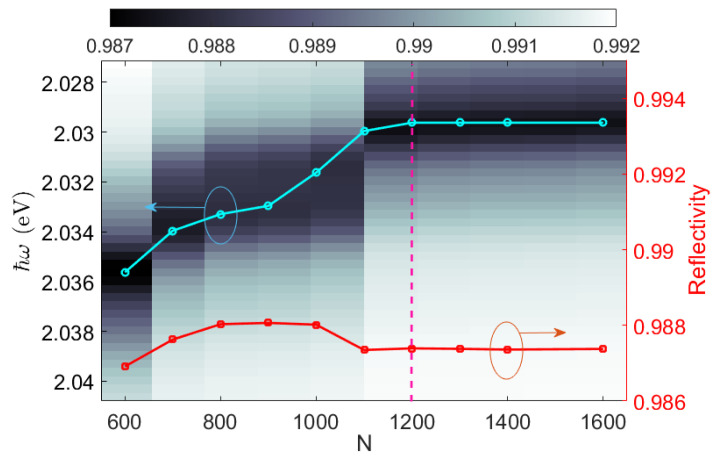
Reflectivity map R(N,ℏω) and spectral position of the BSW resonance (cyan circles) in an exemplary cavity with spacer width W=500 nm and 360 DBR periods overall at $\varphi =0^{o}$ incidence. N indicates the number of Fourier terms retained in the RCWA code. The reflectivity values at the resonance center (red squares) are also plotted. The dashed pink line indicates the number of harmonics used in this work.

Calculations were performed by means of a freely available implementation of the Rigorous Coupled Wave Analysis (RCWA) RETICOLO [52,53]. The RCWA supercell includes the spacer surrounded by 180 DBR periods on each side. The periodic corrugation has an associated Bragg grating vector oriented along the x-axis, i.e., $K_{\mathrm{DBR}}=\left(2\pi \cdot \Lambda_{\mathrm{DBR}}^{-1},0,0\right).$ The conical mounting configuration is considered so that the incoming radiation can be made incident onto the bottom interface of the multilayer at an incident angle θ with respect to the normal (z-axis) and at an angle φ with respect to the x-axis. At $\varphi =0^{o}$, the incident light is parallel to the $K_{\mathrm{DBR}}$ and diffracted accordingly. Illumination is always provided from the glass substrate to reach momentum values beyond the light line in air, as required for BSW coupling. RCWA is a Fourier-based method involving a Rayleigh expansion of the diffracted field as well as a Fourier decomposition of the structure harmonics. For this reason, it is crucial to define the proper number of Fourier terms to be retained in the calculation. The case of the spacer surrounded by DBRs is addressed using N = 1200 Fourier terms. In the convergence plot in Figure 2, the spectral position of the BSW cavity mode and the corresponding reflectivity are shown as a function of the number of Fourier terms considered. Such a supercell contains a number of DBR periods large enough to avoid significant cross-talk effects between adjacent supercells occurring in the momentum–energy region wherein the cavity mode is located. Instead, the simpler case of a purely periodic corrugation (i.e., no spacer) is modeled as an elementary cell containing a single DBR period and N = 60 Fourier terms for assuring reliable results.

**Figure 3:**
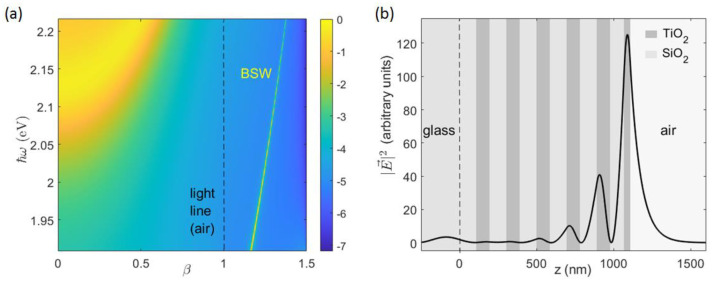
(**a**) Calculated TE−polarized log(1−R(β,ℏω)) of the flat 1DPC, with illumination from the bottom glass substrate at an incidence angle θ such that $\beta =n_{\mathrm{glass}}\cdot \sin\theta$; (**b**) intensity profile of the BSW at ℏω=2 eV, normalized to the amplitude of the incident wave.

The multilayer supports TE-polarized Bloch Surface Waves whose dispersion changes from the near-infrared to the visible spectrum. The dispersion of the BSWs on the flat multilayer can be inferred upon calculation of the reflectivity R(β,ℏω), wherein the effective refractive index is $\beta =n_{\mathrm{glass}}\cdot \sin\theta$ and ℏω is the photon energy of the TE-polarized incident radiation. Without loss of generality, the BSW dispersion can be identified by the pairs $\left(\beta_{\mathrm{BSW}},\omega \right)$ corresponding to the bright line in Figure 3a. The TiO_2_ and SiO_2_ layers have been given a small imaginary refractive index $k_{{\mathrm{SiO}}_{2}}=k_{{\mathrm{TiO}}_{2}}=10^{-3}$ to introduce enough losses to make the BSW dip visible on the calculated reflectivity maps. In Figure 3a,b, the log(1−R(β,ℏω)) map and the normalized intensity profile of the BSW at ℏω=2 eV are respectively shown.

## 3. Results

**Figure 4:**
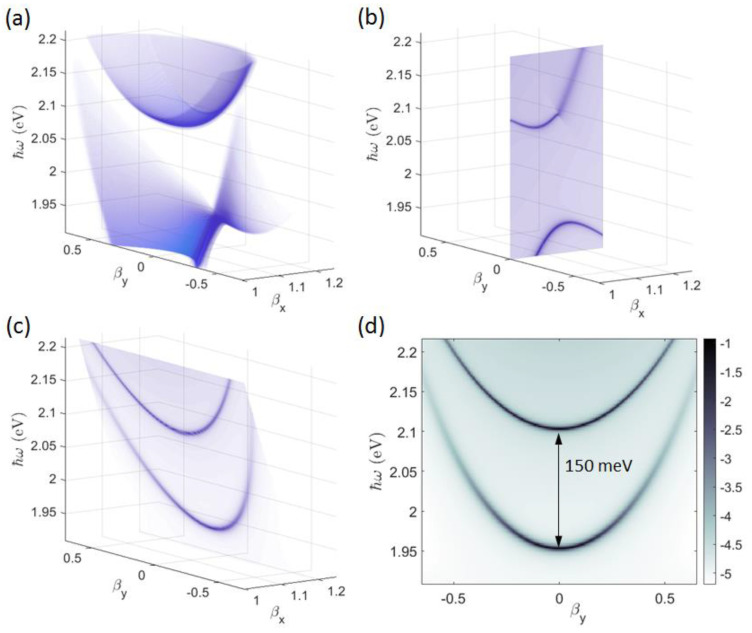
(**a**) Dispersion of a TE−polarized BSW represented as a 3D surface from the calculated reflectivity $R\left(\beta_{x},\beta_{y},\hbar \omega \right)$ of the 1DPC patterned with the periodic DBR. Only the region beyond the light-line in air $\sqrt{\beta_{x}^{2}+\beta_{y\;}^{2}}\geq 1$ is considered; BSW dispersion on (**b**) the $\beta_{y}=0$ plane and (**c**) the boundary of the first Brillouin zone defined by the shaded surface $\sigma =\pi \hbar c\cdot {\left(\Lambda_{DBR}\cdot \beta_{x}\right)}^{-1}$; (**d**) $\log\left(1-R\left(\beta_{x},\beta_{y},\hbar \omega \right)\right)$ map on the σ surface, where $\beta_{x}=\pi c\cdot {\left(\Lambda_{DBR}\cdot \omega \right)}^{-1}$.

The case of the planar stack corrugated with a purely periodic surface modulation (no defect spacer) is considered first. In Figure 4a, the energy and angularly resolved full dispersion of the TE-polarized BSWs is shown as a 3D surface whose points $\left(\beta_{x},\beta_{y},\omega \right)$ are extracted from the calculated reflectivity R(β,φ,ℏω). There, $\beta_{x}=\beta \cdot \cos\varphi$ and $\beta_{y}=\beta \cdot \sin\varphi$, and they must match the BSW effective refractive index at any BSW energy ℏω, i.e., $\sqrt{\beta_{x}^{2}+\beta_{y}^{2}}=\beta_{BSW}\left(\hbar \omega \right)$. Reflectivity values refer to the diffraction efficiency of the 0th order at different illumination conditions.

The periodic modulation along the x-direction results in a folding of the BSW dispersion and the formation of a forbidden band that is dispersed in energy. To facilitate the interpretation, the BSW folding and the corresponding energy gap at$\;\beta_{y}=0$ (corresponding to φ=0, i.e., a direction parallel to the grating vector $K_{DBR}$) is presented in Figure 4b. The folding of the BSW dispersion due to diffraction at the $-1^{\mathrm{st}}$ order results in a gap opening at about 2 eV. As pointed out elsewhere [40,54], the energy gap width depends on the effective refractive index contrast introduced by the periodic modulation. More interestingly, along directions different than $K_{DBR}$, the gap shifts to higher energies. Band edges are identified at the intersection of the surface $\sigma \left(\beta_{x}\right)=\pi \hbar c\cdot {\left(\Lambda_{DBR}\cdot \beta_{x}\right)}^{-1}$ (first Brillouin zone boundary, $K_{DBR}/2$) with the full BSW dispersion, as shown in Figure 4c. The forbidden band can be better appreciated by projecting said BSW dispersion onto the $\left(\beta_{y},\hbar \omega \right)$ plane (Figure 4d). A maximum bandgap of about 150 meV at $\beta_{y}=0$ is found. For larger values of $\left|\beta_{y}\right|$ and higher energies, the bandgap is narrowed down.

**Figure 5:**
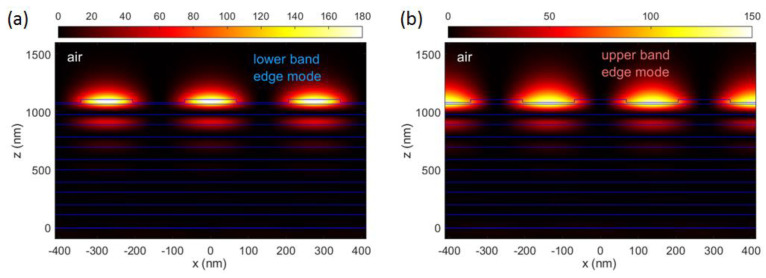
Intensity field distribution $\left|<{\left|E_{y}\left(x,z\right)\right|}^{2}>\right|$ of (**a**) the lower band BSW, calculated at $\hbar \omega_{L}=1.953$ eV, $\beta_{x}=\pi c\cdot {\left(\Lambda_{DBR}\cdot \omega_{L}\right)}^{-1}$, $\beta_{y}=0$; (**b**) the upper band BSW, calculated at $\hbar \omega_{U}=2.1$ eV, $\beta_{x}=\pi c\cdot {\left(\Lambda_{DBR}\cdot \omega_{U}\right)}^{-1}$, $\beta_{y}=0$.

At each bandgap edge, two counterpropagating BSWs are produced, whose interference pattern has a spatial period equal to the DBR period $\Lambda_{\mathrm{DBR}}$. In Figure 5a,b, the normalized intensity distributions of the BSWs at the lower and the upper band are respectively shown. The field localization on either the ridges (high refractive index regions) or trenches (low refractive index regions) reflects the different energies associated to the two band edge modes. Calculations are performed at $\hbar \omega_{L}=1.953$ eV and $\hbar \omega_{U}=2.1$ eV for the lower and the upper band edge modes, respectively. In both cases, $\beta_{x}$ is taken on the boundary of first Brillouin zone, i.e., $\beta_{x}=\pi c\cdot {\left(\Lambda_{DBR}\cdot \omega_{L,U}\right)}^{-1}$, while $\beta_{y}=0$. Field amplitudes are normalized to the incident radiation amplitude.

**Figure 6:**
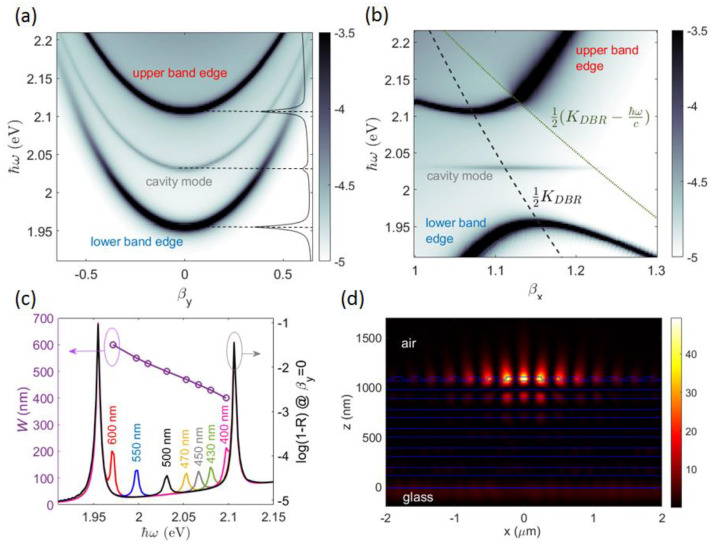
Dispersion of a TE−polarized BSW on the 1DPC patterned with a cavity spacer W=500 nm surrounded by 180 DBR periods on each side, calculated on (**a**) the σ surface, (**b**) the $\beta_{y}$ = 0 plane; (**c**) spectral position (purple circles) and corresponding spectra of the cavity mode across the forbidden band calculated at $\left(\beta_{x},\beta_{y}\right)=\left(\pi c\cdot {\left(\Lambda_{DBR}\cdot \omega \right)}^{-1},0\right)\;$for several spacer values W, i.e. 400 nm (pink line), 430 nm (green line), 450 nm (gray line), 470 nm (yellow line), 500 nm (black line), 550 nm (blue line), 600 nm (red line); (**d**) near-field intensity of the BSW cavity mode ${\left|E_{y}\left(x,z\right)\right|}^{2}\;$for W=500 nm, at ℏω=2.032 eV, $\beta_{y}=0$.

If a linear defect is introduced into the DBR, a cavity mode appears within the forbidden band created by the BSW folding. In Figure 6a, the BSW cavity mode corresponding to a spacer width W=500 nm is visible as a narrow band starting at about 2.03 eV. In comparison to the spacer mode in a conventional planar microcavity, the BSW cavity mode follows a dispersion curve depending on the momentum component that is transverse to the direction of the refractive index modulation. In our case, since the pattern modulation is developing in the x-direction, the BSW cavity mode is energy dispersed as a function of $\beta_{y}.$ It is worth noting that, for$\;\left|\beta_{y}\right|>0.6$, the cavity mode tends to merge with the lower band edge mode, thus loosing spatial localization. Instead, the BSW cavity mode is dispersionless along the $\beta_{x}$ direction (Figure 6b).

The spectral position of the cavity mode can be modulated across the forbidden band by varying the spacer width *W* (Figure 6c). In Figure 6d, a cross-sectional view of the spatial near-field intensity of the BSW cavity mode at ℏω=2.03 eV is illustrated. As in a Fabry–Perot planar microcavity, the energy of the cavity mode is preferentially localized in the defect region. However, the very low refractive index contrast experienced by the BSW prevents a strong confinement within the spacer only, with a significant penetration of the mode profile in the surrounding periodic corrugations.

Very recently, multilayers sustaining BSWs coated with organic/inorganic thin layers exhibiting excitonic resonances have been exploited as novel platforms to enhance light–matter interactions [12,13,55]. In this framework, 2D materials (e.g., Transition Metal Dichalcogenides, orTMD monolayers) are particularly attractive because of the possibility to operate polariton manipulation at room temperature [56,57]. Note that the TE-polarization of BSW facilitates the interaction with excitons whose transition dipole lays on the plane, as occurs, for example, in WS_2_ monolayers [58]. Strong coupling effects between BSWs and excitons have been experimentally studied on flat or periodically patterned multilayers, wherein the resulting polariton field exhibits a spatially delocalized distribution.

**Figure 7:**
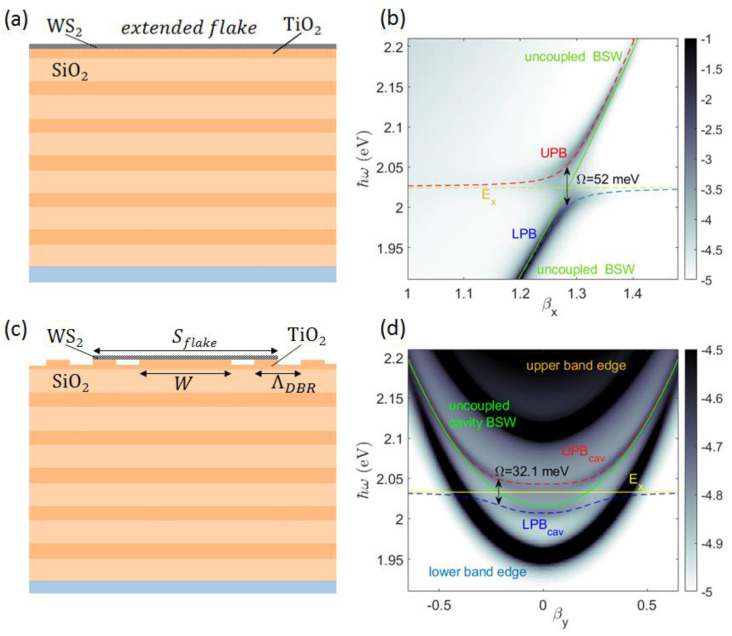
(**a**) Sketch of the z-cross section of the flat 1DPC topped with a uniform WS_2_ monolayer with an indefinite extension; (**b**) corresponding dispersion of BSW polariton; (**c**) sketch of the z−cross section of the 1DPC patterned with the cavity topped with a WS_2_ monolayer. The flake has a lateral size $S_{flake}=1.6\;\mu m$ and a thickness 0.65 nm; (**d**) corresponding dispersion of the BSW cavity polariton branches (UPB_cav_ and LPB_cav_).

When our flat 1DPC model is coated with a tungsten disulfide (WS_2_) monolayer (Figure 7a), the BSW dispersion exhibits the typical anti-crossing behavior illustrated in Figure 7b. The upper and lower BSW polariton branches repel each other via a vacuum Rabi splitting Ω that can be estimated by means of a simple coupled harmonic oscillator model. The system Hamiltonian can be expressed as a 2 × 2 matrix [12] with non-zero off-diagonal elements $H=\left(\begin{array}{cc} E_{BSW} & \Omega /2\\ \Omega /2 & E_{X} \end{array}\right)$, where $E_{X}$ is the exciton energy and $E_{BSW}\left(\beta_{x}\right)\;$is the dispersion of the uncoupled BSW. Hamiltonian eigenvalues are: $E_{up}=\frac{1}{2}\left(E_{BSW}+E_{X}+\sqrt{{\left(E_{BSW}-E_{X}\right)}^{2}+\Omega^{2}}\right);{\;E}_{lp}=\frac{1}{2}\left(E_{BSW}+E_{X}-\sqrt{{\left(E_{BSW}-E_{X}\right)}^{2}+\Omega^{2}}\right),$ which correspond to the dispersions of the Upper and the Lower Polariton Branch (UPB and LPB), respectively. A fitting procedure is used to estimate Ω and $E_{X}$. Worth specifying is that, to consider the dielectric loading effect introduced by the WS_2_ monolayer, the values for $E_{BSW}\left(\beta_{x}\right)$ calculated for the bare 1DPC need be adjusted (redshift) by an additional energy term $E_{0}$ that is also obtained from the fit. In Figure 7b, the blue and the red dashed lines represent the lower and upper polariton dispersion, respectively, the yellow line represents the exciton energy $E_{X}\;$= 2.032 eV, and the green line represents the uncoupled BSW dispersion, redshift-corrected. From the fit, we obtain a Rabi splitting Ω= 52 meV, which is similar to previously published results on a different BSW platform [13]

When the BSW cavity is considered, the BSW–exciton interaction occurs over a spatial region that is substantially limited by the transverse size of the cavity mode. The sketch in Figure 7c shows the case of a WS_2_ monolayer deposited on top of the BSW cavity, extending over the spacer and the first surrounding DBR period, with a total size $S_{flake}=1.05\;\mu m$ and a thickness of 0.65 nm [59]. Similar to cavity modes in a planar microcavity, the cavity BSW hybridizes with the excitonic resonance, resulting in a mode dispersion splitting into upper and lower polariton branches (UPB_cav_ and LPB_cav_), as shown in Figure 7d. The Rabi splitting estimated using the fitting procedure described above is found to be Ω= 32.1 meV. Given the half-widths of the BSW cavity mode $\gamma_{BSW}=$6 meV and the A exciton of WS_2_ $\gamma_{X}=$11 meV [60,61], the condition for the occurrence of strong coupling, i.e., $\Omega >\frac{1}{2}\left(\gamma_{BSW}+\gamma_{X}\right)$, is fulfilled.

**Figure 8:**
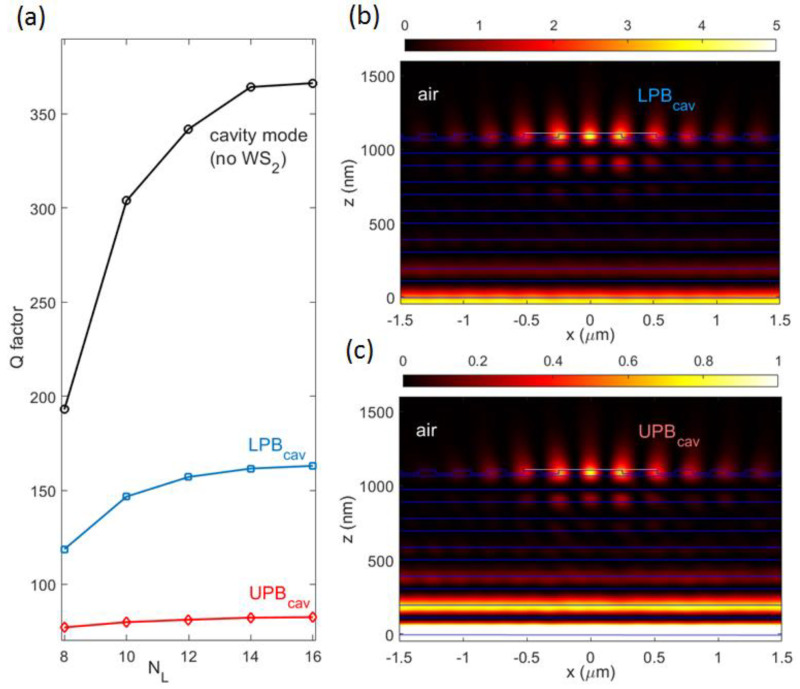
(**a**) Quality factor of upper (red diamonds) and lower (blue squares) BSW cavity polariton branches and the bare cavity mode (black circles) as a function of the overall number of layers N_L_ in the 1DPC; intensity field distribution $\left|<{\left|E_{y}\left(x,z\right)\right|}^{2}>\right|$ of (**b**) the lower cavity polariton LPB_cav_, calculated at $\hbar \omega_{LP}=2$ eV, $\beta_{x}=\pi c\cdot {\left(\Lambda_{DBR}\cdot \omega_{LP}\right)}^{-1}$, $\beta_{y}=0$; (**c**) the upper cavity polariton UPB_cav_, calculated at $\hbar \omega_{UP}=2.04$ eV, $\beta_{x}=\pi c\cdot {\left(\Lambda_{DBR}\cdot \omega_{UP}\right)}^{-1}$, $\beta_{y}=0$. Both intensity distributions refer to an N_L_ = 12 1DPC.

Losses due to the WS_2_ exciton resonance affect the mode quality of the two polariton branches. Figure 8a shows a calculated quality factor Q=ω0δω for the bare BSW cavity mode, UPB_cav_, and LPB_cav_ in multilayers made of 8 to 16 layers. For all three modes, resonance frequencies $\omega_{0}$ are recorded on the corresponding dispersion curve, at $\beta_{y}=0$. While an increase in Q with the number of layers N_L_ is ascribed to a decrease in leaky power tunnelling through the 1DPC, the absorption by WS_2_ is responsible for the lower quality of both polariton branches, as compared to the bare cavity mode. Furthermore, the upper branch polariton is more severely attenuated, as also observed experimentally in an analogous system involving strong coupling between ZnO excitons and BSWs [12]. Intensity distributions calculated for UPB_cav_ and LPB_cav_ at $\beta_{y}=0$ are localized in the neighborhood of the spacer, similar to the bare cavity mode, with a substantially reduced enhancement due to losses (Figure 8b,c).

**Figure 9:**
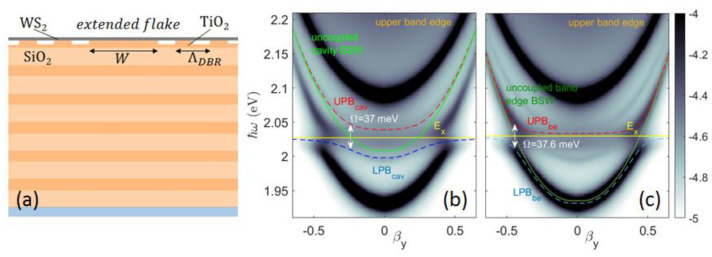
(**a**) Sketch of the z−cross section of the 1DPC patterned with the cavity topped with an indefinitely large WS_2_ monolayer; corresponding dispersions of hybridized (**b**) BSW cavity polariton mode UPB_cav_, LPB_cav_, and (**c**) BSW band edge-polariton mode UPB_be_, LPB_be_.

It is interesting to note that the BSW hybridization presented above involves the cavity mode only. This is due to the limited size of the WS_2_ monolayer, which overlaps mainly with the cavity spacer, where most of the energy is localized. Instead, if the whole patterned 1DPC is topped with an extended monolayer (Figure 9a), the BSW hybridization is found to occur also with the BSW band edge modes, as shown in Figure 9b,c, resulting in upper and lower band edge-polariton branches (UPB_be_ and LPB_be_). Here, we find a Rabi splitting Ω= 37 meV for the BSW cavity polariton, and Ω= 37.6 meV for the lower BSW band edge polariton. The different spatial distribution of the band edge and the cavity modes enables us to further modulate the photonic mode–exciton interaction by controlling the size and position of the WS_2_ monolayer transferred onto the BSW platform.

## 4. Discussion

Fostered by an increasing interest toward surface waves on dielectric photonic platforms, we investigated some aspects of the transverse confinement of BSWs in open Fabry–Perot microcavities. Despite the low effective refractive index contrast produced by the ultra-shallow patterning/modulations of the multilayer surface, BSWs can be resonantly confined within sub-micrometric regions. As an extension of the findings presented here, an omnidirectional confinement on the multilayer plane can be obtained by introducing axis-symmetric corrugations [40] or dielectric ridges [46]. The design of the surface modulation is addressed in conjunction with the multilayer design, as dielectric loading/unloading effects can significantly influence the BSW dispersion, resulting in momentum mismatch with the pattern. In the exemplary cases presented here, we designed an open Fabry–Perot cavity by employing materials and geometries that can be realistically handled in clean-room fabrication processes. The cavity mode follows a parabolic energy dispersion as a function of the transverse in-plane momentum$\;\left(\propto \beta_{y}\right)$, thus suggesting a two-dimensional analogy to the case of 3D stacked planar microcavities. Cavity Q factors generally increase with the number of layers within the 1DPC due to reduced leakage losses. Although this feature may look advantageous when a BSW is near-field coupled from sources directly located on the 1DPC surface, it can be detrimental in cases of prism-coupling from external sources, because very little power is resonantly transferred from the glass substrate to the 1DPC top surface.

One of the main advantages in BSW-based platforms is the possibility of enhancing light–matter interaction on the surface of the photonic structure. This aspect is particularly useful in the framework of 2D materials such as TMD, that are often transferred on planar or structured surfaces upon exfoliation from bulk. Fostered by the potential applications of polariton handling at room temperature, we addressed the hybridization of a BSW cavity mode with the A exciton in a WS_2_ monolayer deposited thereto. Strong coupling is found with both the cavity and the band-edge modes, depending on the spatial overlap with the interacting modal volumes, resulting in vacuum Rabi splitting in the range from 30 meV to 40 meV.

Our findings contribute to the scientific understanding of BSW-based platforms, particularly of polariton control at room temperature. We anticipate future opportunities for embedding TMDs on dielectric multilayers from the perspective of integrated quantum nanophotonic devices [62].

## Data Availability

Not applicable.
